# A Clinical Lecture before the Washington Medical Society

**Published:** 1910-07

**Authors:** Tom A. Williams

**Affiliations:** Washington, D. C., Memb. Corresp. Soc. de Neurel. de Paris; Memb. Corresp. Soc. de Psychol. de Paris; Neurologist to Ephiphany Free Dispensary


### A CLINICAL LECTURE BEFORE THE WASHINGTON MEDICAL SOCIETY.

### APHASIA.

BY TOM A. WILLIAMS, MB., CM., EDIN.,

*Washington, D. C., Memb. Corresp. Soc. de Neurel. de Paris; Memb. Corresp. Soc. de Psychol. de Paris; Neurologist to Ephiphany Free Dispensary.*

WASHINGTON, D. C.

### DYSARTHRIA, APHEMIA, APRASCIA, IDIOGLOSSIC AMNESIS WITH CASES; AND PROGNOSIS, DIAGNOSIS, TREATMENT.

The term aphasia is often so erroneously applied that it is not out of place to discuss some of the disorders of speech often thus misnamed; for there is the greatest therapeutic importance in knowing whether a speech disorder is (1) a permanent organic defect, congenital or acquired, or whether it is (2) a functional disability due to intoxication, oedema, or (3) arising purely from a vicious habit or fixed idea. A resumé of modern knowledge of this subject is the more useful in that the paper of Pierre Manstke Marie of Paris gave to many the impression that the classical views of aphasia were false; and those who have not examined the question are unaware of the fact that the further studies incited by Marie's article have established more firmly than ever the reginal classification of aphasia.

It will be recollected that Marie denied the occurrence of *aphemia* in any form, by which term we denote the inability to utter words although all the muscular movements for doing so can be managed when words are not in question. It is supposed that the *kinesthetic* images are unavailable either by destruction of their centre, the left third frontal convolution, or by interruption of the path connecting this centre with the psycho-motor area concerned with moving the muscles of speech. Marie termed the loss of utterance *anarthria,* that is absence of power to articulate, a most misleading application of a term always used in quite a different clinical sense; for it is applied only to cases which also show disability of the muscles to normally perform other acts as well as that of speech. This is clearly illustrated by the patient now shown:

In her ANARTHRIAL there was a gradual loss of clearness in the utterance of words and in the modulation of sounds, until now she is utterly unable to articulate a single word or to make any but the most elementary sound, in which it is easy to hear that the vocal cords are quite passive. She is truly anarthric; because as you observe, her tongue is almost completely paralyzed; the palate is highly paretic, as is the pharynx; for she is unable to swallow solids at all, and liquids are taken only with difficulty and sometimes flow through the glottis. There is also a slight paresis of the lower facial and jaw muscles, with a tendency to dribbling of saliva. But she is not an aphasic; could the muscles move, she could utter the words she wants, of which she has a perfectly clear motion, just as she has of the act of swallowing although she cannot perform it. Her disability is caused by the very rare condition, the partial necrosis of the homologous areas of the two hemispheres which bi-laterally govern the movements of the tongue, larynx, pharynx, palate and lower part of the face. So far as I know, this case is unique; for no other areas are implicated.

*Dyarthria* is the condition heard in bulbar paralysis. It is there accompanied by atrophy of the muscles of articulation; but pseudo-paralysis resembles the case I have shown; for there is no muscular atrophy but only an upper neurone paralysis arising however from infracortical lesions which are usually multiple and proceed from arterial sclerosis.

The cases of *aphemia* where the third frontal appeared intact are explained by serial sections, which show that there is an interruption in the white fibres of the centrum ovale between F 3 and the temporal lobe (Mahaim, Congress of Geneva). The same observer reported two cases without motor aphasia in which the posterior two-thirds of the insula were destroyed.

That the so-called lenticular zone of Marie has nothing to do with *aphemia* is shown by ten cases which Compte cut in series, in which the lenticular nucleus was free from necrosis, although small areas of destruction of the internal capsule had produced the dysarthria of pseudo-bulbar palsy.

When an aphasia is partial, it is usual for the substantives to disappear before verbs and adjectives, more especially in cases of cerebral tumours of the speech zone. The most likely explanation of this is that most substantives are used less often than the common verbs and adjectives. Besides, it would seem by their use by primitive peoples that the verb and adjective are more deeply rooted in speech, as they are acquired first.

The inability to pronounce certain consonants, or speaking in a nasal voice may be a congenital defect, or fault of training; but the nasal and thick voice quite often occur in the course of a poly-neuritis from degeneration of the nerves to the palate. The prognosis in this case is quite good as a rule.

Idioglossia. The most important element in the prognosis *dysarticulosis of childhood* is the perseverance and intelligence of the mother and nurse. This is illustrated by a musician's child aged four and a half who was under my care in Paris on account of almost unintelligible utterance. I was not sure whether this was entirely due to some congenital defect of the nerve apparatus. Had I given up efforts after the mother had failed in two weeks to produce the least improvement, the former diagnosis might have been too quickly acquiesced in. But as I was pretty sure that training could overcome at least part of the disability, the father was urged to take the child in hand; and his efforts, seconded by those of the mother, completed a cure in four months.

We may pass now to the *treatment of motor aphasia.* The chief difficulty to conquer is the annoyance and loss of mental balance the patient feels in not being able to perform the habitual automatic action of speaking. Thus, *psychotherapy* becomes an important element of the training. A child is accustomed to being controlled and compelled to do things which he dislikes, in the acquisition of arts. But it is very difficult for an adult to turn his energies into new activities. More especially is this the case with the difficult co-ordination required for a new automatic speech apparatus. One of the best methods is the learning of verse and its repetition. Very important is the utilization of the motor images of the lips seen in a mirror, as in the training of deaf-mutes.

The better *prognosis* of aphasia as compared with aphemia is due to the constant re-education the patient's surroundings give. He is assailed all day long with words by hearing or sight, and has to make efforts to comprehend in order to understand his fellows. On the other hand, so difficult are the first words of the aphemic that inertia is apt to get the better of him, and efforts to speak cease to be made. As soon, however, as an improvement begins, each year will bring further gain.

Perhaps the greatest factor in prognosis is the intellectual and moral status of the individual. If the memory is gone, hope is small. If internal language has disappeared as well, the prognosis is bad. Simple methods of testing the internal language are for the patient to indicate the number of syllables in the word representing an object shown him.

In the diagnosis of aphemia it is important:—

(1) To exclude dysarthria.

(2) To exclude sensory aphasia, that is, word-deafness or word blindness.

(3) To test the power of mimicry and the repetition of words. Spontaneous mimicry only disappears as intelligence is impaired.

(4) To test the singing which is often retained along with the vocalization, which in some people is merely an automatic accompaniment of the music.

(5) To test the reading power, which is nearly always at first slightly *alexic,* possibly through the mechanism which Von Monakow has termed *diaschisis.* This may be expressed simply as the interference with the function of one centre on account of the impairment of another centre with which the first is intimately connected physiologically.

(6) Writing with the left hand is sometimes possible; and it may be reversed as in a mirror; and the name can nearly always be signed, though rarely spoken in complete cases.

*Differential diagnosis from sensory aphasia* is easily effected by the transcription of letters, which is made in a servile fashion, and is really a drawing when there is word blindness. *Simulation* is detected by its complete mutism; the real aphasic can always utter some sound, and it is characteristic that he makes most violent attempts to speak, while the simulator remains quite calm and makes no sound at all. The pretended mutes' mimicry is not defective; and he has no difficulty of comprehension, and is not likely to show *apraxia,* unless he has been very well coached; besides, the true aphemic rarely attempts spontaneously to write with the contralateral bend, while a pretended or hysteric readily does so.

A few words on *apraxia,* of which aphemia is merely a special kind. The term is used to denote an inability to perform an act conceived, and the substitution of some other act, an inability neither due to disorientation, amnesia, aprosexia (lack of attention), paralysis, ataxia nor agnosia (inability to recognize objects felt, smelt, heard and seen). It is very simply ascertained by demanding such acts as putting out the tongue, touching the ear, raising the leg, etc., or by the less simple series of acts used in lighting a cigarette, cutting up one's food, and the still less simple acts used in dressing one's self, sealing a letter, etc.

The act of putting a cheque in the waste basket and pocketing the envelope is not strictly of the same order; for it is due to *failure of attention.*

Still another form is characterized by a general contraction of all the muscles of the limb concerned in the desired acts which are less and less well performed as they are more complicated. The order of excellence in Kleist's case was (1) flexion and extension, (2) turning, sitting, arising, (3) going up stairs, standing on one foot, on tip-toe, (4) impossible to leap, to draw a circle in air with foot.

The faculty of performing orderly movements, *eupraxia,* is believed to reside in the left third and second convolutions. This centre guides the co-ordinate purposive movement complexes of limb on both sides. Therefore, the corpus callosum must be intact for its action. Its destruction does not seem to impair the *autokinetisms,* whether these are merely muscular *synergias,* such as the (1) extension of the wrist when the fingers are flexed, (2) the dorsi-flexion of the foot when the leg is lifted, (3) the bending of the knees when the trunk is extended backwards, or whether they are certain *habitual movements* (4) such as walking, (5) shaking hands.

*Sensory aphasia* is really a form either of *amnesia* or of *agnosia,* and as failure of recognition is in reality lack of power to remember either the thing itself or to recall the connections which associate it with other things, amnesia becomes the chief factor in a sensory aphasia of the acquired type.

*Agenesia verbalis.* When congenital, moreover, it is the failure to fix the memories of words which give rise to the word blindness which perhaps occurs to a greater or less degree in about two per thousand school children, who never acquire the power of reading with facility as a result of their school training. The powerful stimulus of some interest in after life, however, may call forth the extra effort needed for such defectives to learn to read freely. Thus one of Hinshelwood's cases (Brit. Med. Jour., 1907, Vol. II) taught himself to read in four years through his interest in the football reports, over which he pored for four hours every evening in oreder to follow the game for which he had a passion. Seven years at school had hardly taught him to read a word.

Patients of this type can in reality always be taught to read if placed in a special class where the tactile images are cultivated by the use of blocked letters. It is believed that the defect is due to hereditary insufficiency of the associational area between the visual centre and the word-hearing centre. We do not know whether their training is made possible through the use of the right-side centre or whether it is not accomplished entirely by the association of kinesthetic images with the sight of the written words, as in the deaf mute.

The condition must be *diagnosed from ocular* defects by (1) the size of the letters making no difference to the reading power of the patient, (2) by the patient's ability to copy the letters, (3) by the fact that figures and some few words and letters are readily recognized. The general mental defect of *imbecility* is excluded by the fact that the child's auditory memory is quite good and that all school tasks except reading are normal.

The same principles of diagnosis and educative treatment may be applied to *adult alexics;* but the prognosis is qualified by the facts mentioned when discussing aphemia, viz.: the difficulty of a mature person to recommence the kind of education which every infant performs as a matter of course.

*Word deafness* may be classified into three degrees: In the most grave, the patient does not know he is listening to speech at all when words are uttered in his presence. In the second, he does not recognize the language; and thirdly, in the slightest degree, he does not understand the meaning: the extent of this failure may vary from complete want of comprehension to an uncertainty or confusion only in complicated sentences.

The *prognosis* of the latter cases is fairly good when the cause can be suppressed; for it would seem that considerable education of the auditory word-centre in the first temporal convolution is possible; and the patient can hardly help automatically listening to the words of his companions.

As the text-books contain clear and full descriptions of this affection I shall not enlarge, except to point out the distinctions between it and the *functional aphasias and amnesias of psychic origin.* The best way to do this is by the citation of cases recently seen.

The first is an example of *aphasic amnesia* from organic causes. It is introduced by way of contrast.

It is characterized by the loss of power to express the thought in words although the patient *feels* that the idea is there, and can sometimes explain it in a periphrasis. My patient, even after ten minutes study, is unable to recall and relate in words the story of Noah Webster. The following is her account with much questioning:

It begins with N. *Noel?* No. *Noah?* No. *Newel?* No. *Noah?* That's like it. *Recognizes Webster.* College? Yale. *Profession?* Lawyer. *What did he do when young?* Taught school. *What else?* Don't know. *A book?* Yes. *Kind?* Don't know. *Dictionary?* I suppose. *Date?* 1790, too late. *Why?* My idea earlier but I don't get the rest. *Was it in war time?* The other one. *Name?* —. .*Against whom?* All other kinds in United States. *Civil?* Yes, that is it. *Date?* 1861. *Did Webster live then?* Too soon. *Against whom then?* He was not old enough, don't remember now though I taught school.

She did not recollect the capital of Germany, nor in what country Edinburgh was situated, although she had once been a school teacher; but she could reply quite intelligently to any question if she remembered the word to answer, and she could read and write, but in the latter there were frequent omissions of words, of which she was quite aware, and deplored it. She could not recall the word she wanted, although she at once recognized it when proffered. Her condition intermitted, more or less, but never became normal. The importance of the case lay in its diagnosis from a purely psychic amnesia; and to ascertain whether it was not due to a chronic intoxication, or whether it was caused by the cerebral necrosis of arterial defect, as I finally decided, as metabolic treatment failed to ameliorate the condition, and its oscillations were very slight. That it was not a psychic case is shown by its entire unamenability to psychotherapy, by its genesis apart from any psychological cause, and by the nature and extent of the responses to psychometric tests too long to detail here.

The second case, seen with Dr. Clayton at the Garfield Hospital, is an example which may be called *hysterical aphasia* in which the fixed idea not to speak was a *reaction of defense* against the fear of relating some intensely humiliating experiences. Indeed, the patient tried to chase these from his thoughts, and the aphasia was partly in consequence of this. It is comparable to the reaction of one of Janet's patients, who, because she wished to conceal a fault from everyone, began to lie not only about that but about everything that she spoke.

Another case which reacted in this way was conduced too by aphasic as a result of trying to conceal thought. The mechanism of a remarkable case described by Dr. Hickling is similar; but is derived from a fixed idea, which is only part of a general paranoid trend. The patient becomes aphasic on account of the delusion that he has been ordered not to speak for so many hours by his hypnotiser.

Formerly, the psychological mechanism of hysterical aphasia was a mystery; but modern *psycho-analysis* has taught us the why and wherefore. Case No. 2 is a very common type.

Another case which reacted in this way was conduced to by a metabolic defect which led also to *narcoleptic confusion*. Complete deafness and aphonia was the result of a most painful experience in a hospital in Idaho. The condition was recovered from quite suddenly by the stimulating touch of a detective.

CEREBRAL TUMOURS AS CAUSES. FAULTY DIAGNOSIS.

Hysteroneurasthenia is quite commonly diagnosed to explain paraphasia which later proves to be due to a cerebral tumour. Such was the case of the girl whom I observed in Paris, and who was treated as a neurotic and isolated for several months by so eminent a neurologist as Déjerine. In the autumn she entered the clinic of Ballet with a well-marked amnesic aphasia for substantives, very similar to that of the first case here reported. She had all along been inattentive and frivolous in her ways and had shown slight mental dullness. I watched the slow development of the papilloedema, which clinched the diagnosis and a tumour was found in the left temporo-parietal sub-cortex.

Such recurring attacks of aphasia must be distinguished from those due to opthalmic migraine; for both may be accompanied by headache and vomiting. The jargon-aphasia of opthalmic migraine is, however, almost always accompanied by hemianopsia, and the attacks are of very short duration; and separated by a period of complete good health, whereas a careful examination of these tumour cases will reveal a defect of speech even between the exascerbations.

In conclusion, this sketch of a vast and complicated subject is designed to emphasize the extreme importance of early diagnosis of forms of aphasia which are purely psychic and which are most amenable to proper treatment when taken early. Even in the organic forms early detection of the cause may enable us to impose means for its suppression and thus to arrest even though not to cure the patient's disease. Such are the cases of temporary aphasia which occur in the course of arterial sclerosis through the variations of blood pressure and osmosis in the weakened blood vessels. The oedemas of uremic states are of this variable type too.

Lastly, variable amnesia and slow comprehension may be one of the earliest signs of intracranial new growth, which may be revealed in an examination by one skilled in neurological technique.

I have said nothing of the defects of speech arising from faulty habits, of mental attitudes, such as stutterings, lolling, lisping and the tics of speech, coprolalia, etc., in which the patient is compelled to utter obscene remarks, or barking or coughing. These are psychasthenic phenomena and form a chapter of neuropathology in themselves.

REFERENCES.

1. Semaine Med., 1906.

2. Déjerine Presse Med., 1906-1907. Also Discussion sur l'Aphasie, Rev. Neurol, 1908, etc.

3. Thése de Paris, 1900. Les Paralysies Pseudo-bulbaire.

4. Author, The Trend of the Clinical Concept of Hysteria. Boston Med. Jour., 1909, March.

6. See Considerations as to the Nature of Hysteria. International Clinics, 1908, Vol. III.

